# Burden of moderate to severe anaemia and severe stunting in children < 3 years in conflict-hit Mount Cameroon: a community based descriptive cross-sectional study

**DOI:** 10.1186/s12887-020-02296-2

**Published:** 2020-08-24

**Authors:** Irene Ule Ngole Sumbele, Gillian Nkeudem Asoba, Rene Ning Teh, Samuel Metuge, Judith Kuoh Anchang-Kimbi, Theresa Nkuo-Akenji

**Affiliations:** 1 Department of Zoology and Animal Physiology, University of Buea, Buea, Cameroon; 2 Department of Microbiology and Immunology, Cornell College of Veterinary Medicine, Ithaca, New York USA; 3 Department of Social Economy and Family Management, Higher Technical Teachers’ Training College, University of Buea, Kumba, Cameroon; 4 Department of Microbiology and Parasitology, University of Buea, Buea, SW Region Cameroon

**Keywords:** Anaemia, Armed conflict, Children, Feeding habit, Malaria parasite, Moderate to severe anaemia, Microcytic anaemia, Microcytosis, Severe stunting, Undernutrition

## Abstract

**Background:**

Armed conflict is a significant social determinant of child health with nuanced effects. There is a dearth of knowledge on the public health issues facing vulnerable populations in conflict-stricken areas. The objective was to determine the prevalence and determinants of moderate to severe anaemia (M*d*SA) and severe stunting (SS) in children ≤3 years in conflict-hit Dibanda, Ekona and Muea in the Mount Cameroon area.

**Methods:**

Haematological parameters were obtained using an automated haematology analyser while undernutrition indices standard deviation (SD) scores (z- scores), were computed based on the WHO growth reference curves for 649 children in a community based cross-sectional study in 2018. Binomial logistic regression models were used to evaluate the determinants of M*d*SA and SS against a set of predictor variables.

**Results:**

Anaemia was prevalent in 84.0% (545) of the children with a majority having microcytic anaemia (59.3%). The prevalence of M*d*SA was 56.1% (364). Educational level of parents/caregiver (*P* <  0.001) and site (*P* = 0.043) had a significant negative effect on the occurrence of M*d*SA. Stunting, underweight and wasting occurred in 31.3, 13.1 and 6.3% of the children, respectively. Overall, SS was prevalent in 17.1% (111) of the children. The age groups (0.1–1.0 year, *P* = 0.042 and 1.1–2.0 years, *P* = 0.008), educational levels (no formal education, *P* <  0.001 and primary education *P* = 0.028) and SS (*P* = 0.035) were significant determinants of M*d*SA while M*d*SA (P = 0.035) was the only significant determinant of SS. On the contrary, age group 0.1–1 year (OR = 0.56, *P* = 0.043) and site (Dibanda, OR = 0.29, *P* = 0.001) demonstrated a significant protective effect against SS.

**Conclusions:**

Moderate to severe anaemia, severe stunting and wasting especially in children not breastfed at all are public health challenges in the conflict-hit area. There is a need for targeted intervention to control anaemia as well as increased awareness of exclusive breast feeding in conflict-hit areas to limit the burden of wasting and stunting.

## Background

Armed conflict is a public health concern [1]. The violent and destructive nature of armed conflicts and the breakdown in health systems may harm vulnerable populations like children under 5 years and pregnant women residing in such areas who themselves are rarely combatants. A significant portion of child deaths in Africa take place in countries with recent history of armed conflict and political instability. Approximately 4·9–5·5 million deaths of children younger than 5 years between 1995 and 2015 were related to armed conflict [2, 3]. Cameroon, a country once known for its stability, has faced violence in an armed conflict since 2017 with serious human rights abuses and humanitarian consequences of great concern in the North West and South West Regions [4]. Armed conflict is a significant social determinant of child health with nuanced effects on physical, developmental, mental health and wellbeing [5].

Exposure to armed conflict is associated with a higher burden of infectious disease in children such as malaria [6], with anaemia as a common or sometimes serious complication. Childhood anaemia is an important outcome indicator of the burden of malaria, poor nutrition and health and, could be considered as a marker of socio-economic disadvantage as the poorest and least educated are at the greatest risk of exposure to its risk factors and sequelae [7]. It is a major public health problem globally in children under 5 years with an estimated prevalence of 47% [8]. In Cameroon, the prevalence of anaemia in children 6 months to 3 years ranges from 66.6–83.6% [9]. Following intervention studies in the Mount Cameroon area in 2006, the prevalence of anaemia in children less than 5 years dropped from 84.1 to 37.9% in 2013 [10]. Anaemia in childhood may lead to delayed growth, impaired cognitive and behavioural development as well as morbidity such as increased susceptibility to infections [11–13] while, severe anaemia has been reported as a significant cause of mortality [14].

Defined as a decreased concentration of haemoglobin (Hb) that leads to reduced capacity for oxygen transportation, anaemia may be classified as microcytic, normocytic or macrocytic based on the size of red blood cells (RBC) as measured by the mean corpuscular volume (MCV). The level of decrease in concentration of haemoglobin could be categorized as mild, moderate, and severe anaemia. The monitoring of moderate-to-severe anaemia (M*d*SA) is recommended for disease surveillance in countries with high prevalence of malaria and anaemia [7, 15]. The prevalence of *Plasmodium* parasitaemia in children in Cameroon varies from 7 to 85% [16] hence, the need for constant monitoring of the burden of M*d*SA and other nutrition related morbidities is invaluable.

Undernutrition measured by anthropometry is evaluated in outcome variables like stunting, underweight and wasting. Stunting in young children, which represents failing growth, is a consequence of long term, cumulative inadequacies of health and nutrition [17, 18]. The occurrence of undernutrition in the first 1000 days of a child’s life can be very critical with irreversible consequences on the child’s growth as this is a phase during which rapid physical and mental development occurs [19]. Demographic and health surveys between 2006 and 2016 revealed the prevalence of stunting in children under 5 years in sub Saharan Africa was 33.2%, wasting was 7.1% and underweight was 16.3%. In Cameroon, the prevalence of stunting, wasting and underweight was respectively 32.5, 5.6 and 14.6% [20]. Even though Cameroon is not among the vulnerable countries for urgency for strategic interventions aimed at improving child nutrition, the ongoing armed conflict in different regions of the country (Boko Haram in the North, incursions in the East Region and the anglophone crisis in the Northwest and South West regions) increases the vulnerabilities of children living in such areas. There is a scarcity of knowledge on the public health issues facing vulnerable populations in conflict-stricken areas hence, the need for setting-specific information to develop effective anaemia and undernutrition control programmes. The objective of this study was to determine the prevalence and determinants of M*d*SA and SS in children ≤3 years in conflict-hit Dibanda, Ekona and Muea in the Mount Cameroon area.

## Methods

### Study site

The three semi-rural communities of Didanda, Ekona and Muea located at the foot of Mount Cameroon have been adequately described by Asoba et al. [21]. These areas have experienced unrest and clashes between the armed separatist movement and government forces following the Anglophone crisis in the English-speaking regions of Cameroon since 2017 [4]. Ekona, a once vibrant community is amongst the hardest hit areas by the violence and its plantations have been abandoned. Inhabitants in these areas have become internally displaced and it has increasingly turned out to be difficult for the majority of whom are farmers and petit traders to carry out their activities.

### Study design

This community-based descriptive cross-sectional study was carried out between the months of March and October 2018.

### Study participants

The study participants included children between the ages of 1 month and 3 years resident in Dibanda, Ekona and Muea whose parents/caregivers consented to their participation in the study. Children were enrolled in the study if symptoms of cerebral malaria, HIV/AIDS, Kwashiorkor, Sickle cell anaemia and other severe febrile conditions requiring hospitalizations were excluded.

### Sample size and sampling technique

The minimum sample size required for the study was estimated from the previous prevalence of anaemia in malaria parasite positive and undernourished children (43.9%) in the community of Muea [22] using the formula *n* = z^2^pq/d^2^ [23] where *n* = the sample size required, z = 1.96: confidence level test statistic at the desired level of significance, *p* = 0 439: proportion of anaemia prevalence, q = 1-p: proportion of non-anaemic children and d = 0.05: acceptable error willing to be committed. A minimum sample size of 378 was obtained.

The method of sampling involved a multistage cluster sampling in the communities where in the first stage, 3 conflict hit communities were randomly selected from the 29 communities in the Mount Cameroon area. In the second stage, 32 clusters were randomly selected within the three communities. In each of the clusters, children 1 month and 3 years old in all the households were selected until the desired sample size was attained. At the onset of the study, the community was educated on the purpose and benefits of participating in the study. The study team embarked on data collection upon obtaining Administrative authorization and ethical approval for the study.

### Data collection

Data collection sites in each community were identified and organization as well as coordination for the collection of samples was carried out with the aid of local chiefs, block heads and community relay agents. Potential participants were invited for sample collection on specific dates in each community. Upon obtaining consent/assent from the participants, semi-structured questionnaire on socio-demographic and infant feeding practices was administered. Due to the very young ages of the children, parents/caregivers were the respondents. Data on socio-demographics (gender and age of children), feeding habits (exclusive breastfeeding and duration/ mixed feeding/no breastfeeding), types of local weaning foods, history of fever in the preceding 2–3 days, mosquito bed net use, marital status and educational level were obtained. Infants were classified as being exclusively breastfed (EBF) when fed only breast milk for the first 6 months [24]. An infant was considered as having mixed feeding (MF) when he/she had a combination of breast milk and local infant formulae before 6 months while no breast feeding (NBF) infants were those not given breast milk at all from birth and were fed with local infant formula.

The axillary body temperature of each child was measured using an electronic thermometer and fever was defined as temperature ≥ 37.5 °C. Anthropometric measurements which included height and weight were measured using a measuring tape and a beam balance (Terraillon, Paris) while the ages of the children were obtained from their mothers/caregivers and/ or birth certificates. Undernutrition indices which comprised of height-for-age (HA), weight-for-age (WA), and weight-for-height (WH) standard deviation (SD) scores (z- scores) were computed based on the World Health Organisation (WHO) growth reference curves using the WHO AnthroPlus for personal computers manual [25]. Approximately 2–3 mL of venous blood sample was collected from each child using sterile syringes into labelled ethylenediaminetetraacetate (EDTA) tubes and transported to the University of Buea, Malaria Research Laboratory for malaria parasite identification and a full blood count assessment.

### Laboratory procedure

Thick and thin blood films prepared on the same slide and air-dried in the field was fixed in absolute methanol (thin film only), stained in 10% Giemsa for 20 min and examined in the laboratory following standard procedure for the detection, identification and estimation of malaria parasites [26]. Malaria parasite density was determined based on the number of parasites per 200 leukocytes on thick blood film with reference to participants’ white blood cell (WBC) count obtained from the full blood count analysis. Malaria parasitaemia was categorised as low (< 1000 parasites/μL of blood), moderate (1000–4999 parasites/μL of blood), high (5000–99,999 parasites/ μL of blood), and hyperparasitaemia (≥100,000 /μL of blood). Asymptomatic malaria parasitaemia (AMP) was defined as the presence of *Plasmodium* with an axillary temperature of < 37.5 °C [10].

An auto-haematology analyser (MINRAY 2800 BC) was used to assess haematological parameters such as WBC, red blood cell (RBC) and platelet counts, haemoglobin (Hb) level, haematocrit (Hct), mean corpuscular volume (MCV), mean corpuscular Hb (MCH) mean corpuscular Hb concentration (MCHC) and red cell distribution width coefficient of variation (RDW-CV) following the manufacturer’s instructions. The Hb measured was used to define the status of anaemia based on the WHO reference values for age or gender [27].

### Definitions of outcomes

A child was identified as being undernourished if he or she scored <− 2 SD in one of the anthropometric indices of HA (stunting), WA (underweight) and WH (wasting) indices, while corresponding z-scores of <− 3 SD were considered indicative of severe under-nutrition [28]. The public health burden of the forms of undernutrition were interpreted based on the following prevalence ranges; for stunting; low (< 20%); medium (20–29%), high (30–39%) and very high (≥40%); for wasting; acceptable (< 5%), poor (5–9%); serious (10–14%) and critical (≥15%) while underweight was low(< 10%), medium (10–19%), high (20–29%) and very high (≥30%) [18].

The condition of anaemia is defined as Hb < 11.0 g/dL [26] and further categorized as severe (Hb < 7.0 g/dL), moderate (Hb between 7.0 and 10.0 g/ dL), and mild (Hb between 10.1 and < 11 g/dL) [26]. Moderate to severe anaemia is defined as Hb < 10 g/dL, microcytosis as MCV < 67 fL in children under 2 years of age and < 73 fL in children 2 to 5 years of age [27]. Microcytic anaemia is defined as Hb < 11.0 g/dL and presence of microcytosis. Hypochromasia is defined as a MCHC of < 32 g/L [28] and thrombocytopenia as platelet count < 150,000/μL. With respect to anaemia, the following categories were used to interpret the prevalence regarding the public health burden; Severe; > 40%; moderate: 20.0–39.9%; mild: 5.0–19.0% and normal: ≤4.9% [27]

### Statistical analysis

A descriptive data analysis was conducted to describe the characteristics of the study population. The proportions of each factor obtained were compared across the sex and age categories with the use of Chi square (χ^2^) test while the means were compared with the use of t-test and analysis of variance (ANOVA) respectively. Association between the outcome variables of M*d*SA and SS and the predictor variables of age, sex, site, educational level of parent/caregiver and microcytic status were determined using a binomial logistic regression model analysis. The interaction among confounders was also examined. Odd ratios (OR) and 95% confidence interval (CI) were computed and significant differences set at *P* <  0.05. IBM-Statistical package for Social Sciences (SPSS) version 21 was used in the analysis.

### Ethics statement

Administrative clearance was obtained from the South West Regional Delegation of Public Health while, the institutional review board hosted by the Faculty of Health Sciences, University of Buea issued the ethical clearance document (2018/004/UB/FHS /IRB). The protocol was explained and the benefits of participating in the study highlighted to potential participants during the sensitization at the onset of the study. Informed consent/assent forms were presented or read and explained to parents or caregivers of the children at presentation. The consent/assent forms further stated the purpose and benefits of the study as well as the amount of blood to be collected from each child. Only participants who returned a signed consent/assent form and or gave a verbal consent were enrolled in the study. Participation in the study was strictly voluntary. All cases of malaria and those with moderate to severe anaemia or undernourished were referred to the nearest health centre for appropriate treatment and follow up.

## Results

### Characteristics of study participants

A total of 649 children with a mean (SD) age of 1.8 (0.1) years of both sexes (male = 49.6% and female = 50.4%) were enrolled in the study. A greater proportion of the children were from the Ekona semi-rural community (42.1%) and the practice of mixed feeding (MF) by parents was common (60.6%). Majority of the parents/ caregivers had no formal education (43.1%) as shown in Table 1. The prevalence of fever, malaria parasite (MP), asymptomatic malaria parasitaemia (AMP) and hypochromasia were 5.5, 29.4, 27.7 and 6.0% respectively with no statistically significant differences in prevalence by sex and age. The prevalence of microcytosis (70.9%) was common among the children with a significantly higher (*P* <  0.001) occurrence in children 2.1–3.0 years (83.3%) of age when compared with the other age groups. Overall, thrombocytopenia was prevalent in 21.3% of the children with a statistically significant higher presence in those 2.1–3.0 years old (Table 1). The mean Hb level was significantly higher in males (9.5 (1.5) g/dL) while, females had significantly higher Hct (26.6 (4.0) %) and RBC counts (4.1 (1.0) × 10^12^/L) than their respective counterparts (Additional file 1).

**Table 1:** Prevalence of socio-demographic and clinical characteristics of participants by age and sex

Parameters		Sex		*P* -value	Age group in years			Total	*P* value
		Male	Female		0.1–1.0	1.1–2.0	2.1–3.0		
N (%)		322 (49.6)	327 (50.4)		206 (31.7)	222 (34.2)	221 (34.1)	649 (100)	
Site	Dibanda	23.3 (75)	26.3 (86)	0.639	22.3 (46)	27.9 (62)	24.0 (53)	24.8 (161)	0.280
	Ekona	43.5 (140)	40.7 (133)		44.7 (92)	43.2 (96)	38.5 (85)	42.1 (273)	
	Muea	33.2 (107)	33.0 (108)		33.0 (68)	28.8 (64)	37.6 (83)	33.1 (215)	
Infant feeding habit	EBF	20.5 (66)	20.8 (68)	0.939	17.5 (36)	24.3 (54)	19.9 (44)	20.6 (134)	0.368
	MF	61.2 (197)	59.9 (196)		62.1 (128)	59.9 (133)	59.7 (132)	60.6 (393)	
	NBF	18.3 (59)	19.3 (63)		20.4 (42)	15.8 (35)	20.4 (45)	18.8 (122)	
Educational level	No formal	47.8 (150)	38.6 (123)	**0.017**	41.7 (85)	44.9 (97)	42.7 (91)	43.1 (273)	0.859
	Primary	24.2 (76)	24.5 (78)		26.0 (53)	21.8 (47)	25.4 (54)	24.3 (154)	
	Secondary	24.2 (76)	28.2 (90)		26.5 (54)	25.5 (55)	26.8 (57)	26.2 (166)	
	Tertiary	3.8 (12)	8.8 (28)		5.9 (12)	7.9 (17)	5.2 (11)	6.3 (40)	
Prevalence of fever (n)		5.0 (16)	6.1 (20)	0.523	3.8 (8)	5.0 (11)	7.7 (17)	5.5 (36)	0.204
Prevalence of MP		30.4 (98)	28.4 (93)	0.577	32.0 (59)	26.6 (59)	29.9 (66)	29.4 (191)	0.457
Prevalence of AMP		28.3 (89)	27.2 (89)	0.600	30.1 (62)	25.2 (56)	28.1 (62)	27.7 (180)	0.803
Prevalence of microcytosis		73.3 (236)	68.5 (224)	0.179	57.3 (118)	71.2 (158)	83.3 (184)	70.9 (460	**< 0.001**
Prevalence of hypochromasia		4.3 (14)	7.6 (25)	0.77	7.8 (16)	6.3 (14)	4.1 (9)	6.0 (39)	0.269
Prevalence of thrombocytopenia		20.8 (67)	21.7 (71)	0.778	17.0 (35)	18.5 (41)	28.1 (62)	21.3 (138)	**0.009**

*AMP* Asymptomatic malaria parasitaemia, *EBF* Exclusive breastfeeding, *MF* Mixed feeding, *MP* Malaria parasite, *NBF* No breast feeding. *P*- values in bold are statistically significant

### Anaemia prevalence and type

**Fig. 1:**
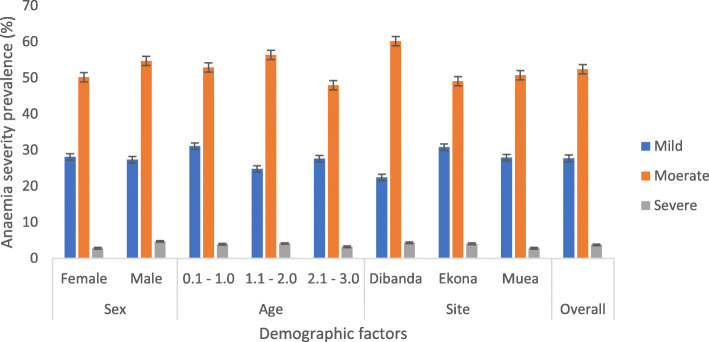
Prevalence of mild, moderate and severe anaemia as affected by sex, age and site

Anaemia was prevalent in 84.0% (95% CI = 81.0–86.6%) of the children. Socio-demographic factors that significantly affected the prevalence of anaemia include age, sex and educational level where, children 0.1–1 year (88.3%), males (87.0%) and children whose parents had no formal education (98.2%) had the highest prevalence. Clinical factors did not significantly affect the incidence of anaemia as shown in Table 2. Majority of the children had microcytic anaemia (59.3%) that was significantly higher in males (64.6%); children whose parents had primary level of education (63.6%); those undernourished (64.3%) and those stunted (66.2%) when compared with their coequals (Table 2). The most common form of anaemia was moderate anaemia (52.4%) with the highest occurring in males, those 1.1–2.0 years old and infants from Dibanda as shown in Fig. 1.

**Table 2:** Prevalence of anaemia and microcytic anaemia as affected by socio-demographic and clinical parameters

Characteristics	Category	N	Prevalence of anaemia	*P*-value	Microcytic anaemia	*P* value
All	All	649	84.0 (545)		59.3 (385)	
Socio-demographic						
Age group in years	0.1–1.0	206	88.3 (182)	**0.022**	53.4 (110)	0.072
	1.1–2	222	85.1 (189)		59.9 (133)	
	2.1–3	221	78.8 (174)		64.3 (142)	
Sex	Female	327	81.0 (265)	**0.040**	54.1 (177)	**0.007**
	Male	322	87.0 (280)		64.6 (208)	
Site	Dibanda	161	87.6 (141)	0.270	59.6 (96)	0.988
	Ekona	273	83.9 (229)		59.0 (161)	
	Muea	215	81.4 (175)		59.5 (128)	
Educational level of parent/caregiver	No formal	273	98.2 (268)	**< 0.001**	70.3 (192)	**< 0.001**
	Primary	154	90.3 (139)		63.6 (98)	
	Secondary	166	63.3 (105)		41.6 (69)	
	Tertiary	40	45.0 (18)		40.0 (16)	
Infant feeding habit	NBF	122	86.1 (105)	0.761	52.5 (64)	0.182
	EBF	134	82.8 (111)		63.4 (85)	
	MF	393	83.7 (329)		60.1 (236)	
Clinical factors						
Febrile status	Febrile	36	83.3 (30)	0.914	63.9 (23)	0.566
	Afebrile	613	84.0 (515)		59.1 (362)	
Malaria parasite status	Positive	191	84.8 (162)	0.706	55.0 (105)	0.145
	Negative	458	83.6 (383)		61.0 (280)	
Undernourished	Yes	249	87.2 (217)	0.082	64.3 (160)	**0.043**
	No	400	82.0 (328)		56.3 (225)	
Stunted	Yes	210	87.6 (184)	0.080	66.2 (139)	**0.014**
	No	439	82.2 (361)		56.0 (246)	
Wasted	Yes	41	92.7 (38)	0.116	65.9 (27)	0.379
	No	608	83.4 (507)		58.9 (358)	
Underweight	Yes	85	87.1 (74)	0.406	65.9 (56)	0.187
	No	564	83.5 (471)		58.3 (329)	

*EBF* Exclusive breastfeeding, *M*F Mixed feeding, *NBF* No breast feeding. *P*- values in bold are statistically significant

### Moderate to severe anaemia (M*d*SA)

**Fig. 2:**
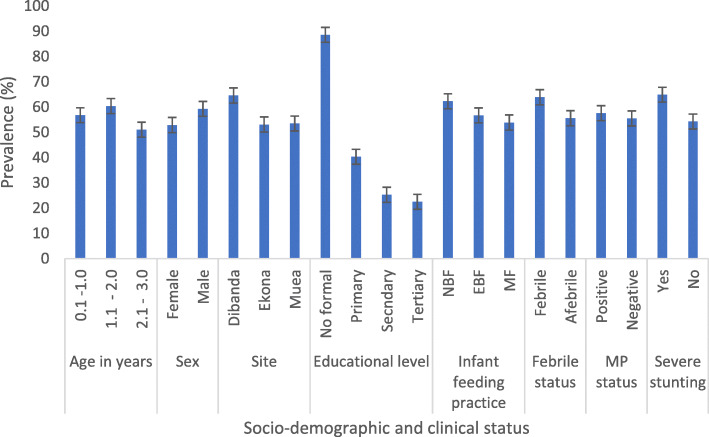
Prevalence of moderate to severe anaemia (Hb ≤ 10 g/dL) by socio-demographic and clinical factors

The prevalence of M*d*SA was 56.1% (364, 95% CI = 52.2–59.9%). Among the socio-demographic factors, the educational level of parents/caregivers (*P* <  0.001) and site (*P* = 0.043) had a significant negative effect on the occurrence of M*d*SA. Children whose parents had no formal education and were from the Dibanda community had the highest prevalence of M*d*SA (88.6 and 64.6% respectively) when compared with the other levels of education and site. Although not significant the prevalence of M*d*SA was highest in children 1.1–2.0 years old (60.4%), males (59.3%) and those who had NBF (62.3%) as shown in Fig. 2. With respect to clinical status, the prevalence of M*d*SA was significantly higher (*P* = 0.041) in children with severe stunting (64.6%) only even though, those febrile (63.9%) and children with MP (57.6%) had a higher prevalence as well than their counterparts (Fig. 2).

### Undernutrition and its forms

**Fig. 3:**
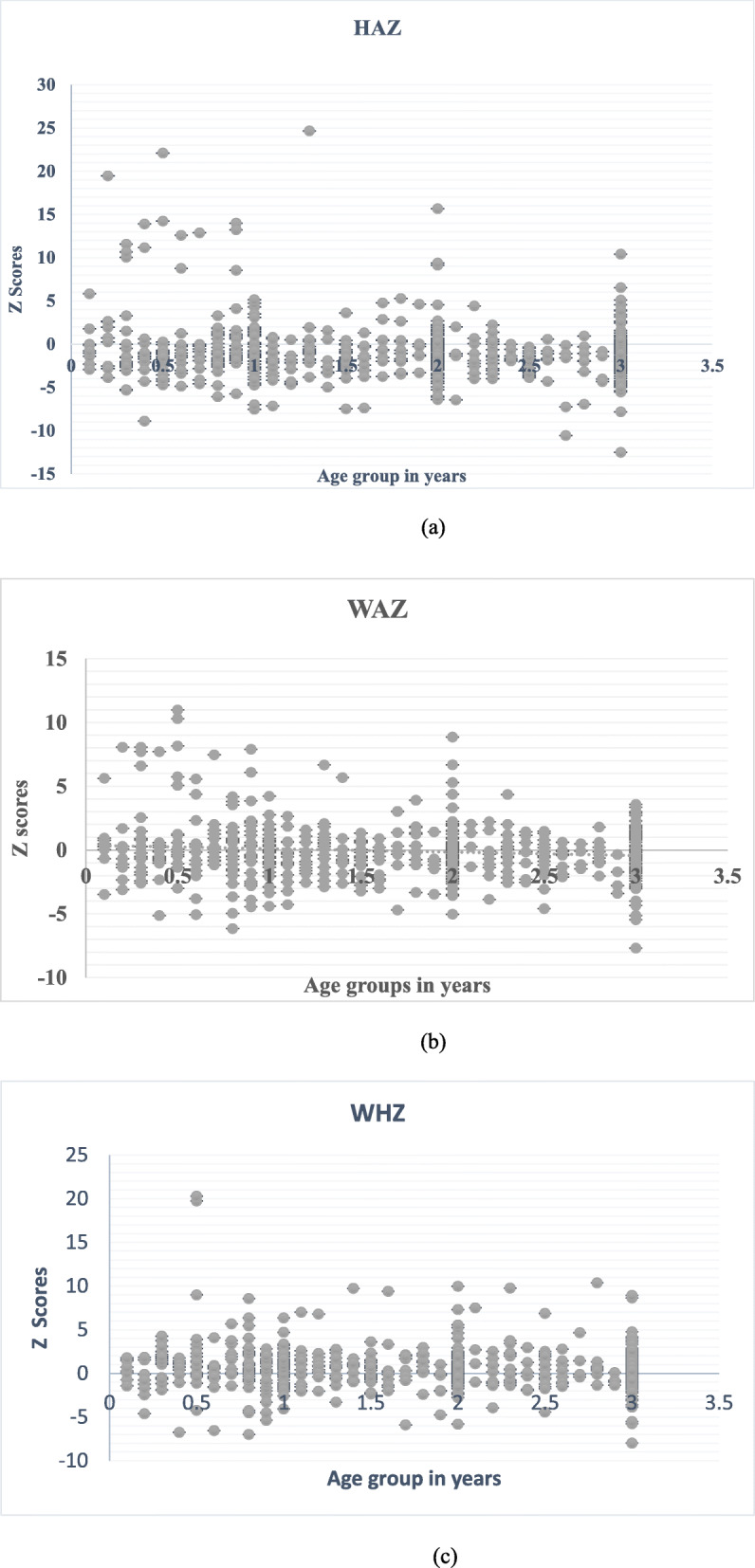
Distribution of z scores of HA (**a**), WA (**b**) and WH (**c**) by age in the study population. HA = Height-for age, WA = weight-for-age, WH = weight-for-height

The distribution of HA, WA and WH z-scores is shown in Figs. 3 (a), (b) and (c). The majority of HA (74.6%) and WA (55.0%) z-scores were in the negatives. The prevalence of undernutrition in the study population was 38.4% (95% CI = 34.7–42.2%). Stunting, underweight and wasting occurred in 31.3, 13.1 and 6.3% of the children, respectively. The prevalence of stunting was significantly higher in children from the Ekona community (41.0%) and those anaemic (33.2%) than their respective equivalents (Additional file 2).

### Severe stunting (SS)

**Fig. 4:**
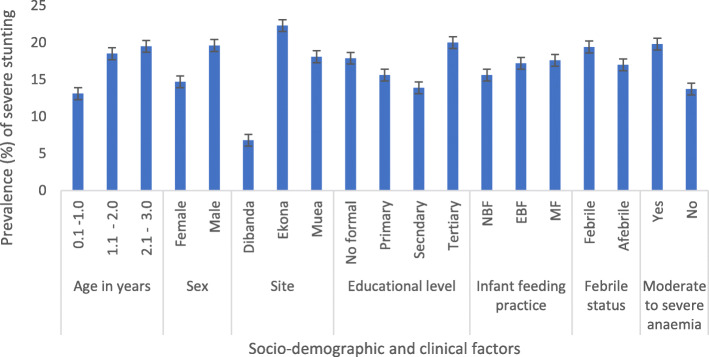
Prevalence of severe stunting by sociodemographic and clinical status

Overall, SS was prevalent in 17.1% (111, 95% CI = 14.4–20.2%) of the children. The prevalence of SS was highest in children 2.1–3.0 years old (19.5%), males (19.6%), those from Ekona (22.3%), children whose parent/caregiver had tertiary education (20.0%), had MF (17.6%), were febrile (19.4%) and had M*d*SA (18.3%) than their respective counterparts. However, only the difference in prevalence of SS by site (*P* <  0.001) and status of M*d*SA (0.041) were statistically significant (Fig. 4).

### Determinants of MdSA and SS

The binomial logistic regression model revealed the age groups (0.1–1.0 year, *P* = 0.042 and 1.1–2.0 years, *P* = 0.008), educational levels (no formal education, *P* <  0.001 and primary education *P* = 0.028) and SS (*P* = 0.035) as significant determinants of M*d*SA. Children whose parent had no formal/ primary education were 32.8 and 2.6 times at odds of having M*d*SA than their counterparts as shown in Table 3. The only significant determinant of SS was M*d*SA (P = 0.035). On the contrary, based on the odd ratios the age group 0.1–1 year (OR = 0.56, *P* = 0.043) and site (Dibanda, OR = 0.29, *P* = 0.001) demonstrated a significant protective effect against SS (Table 3).

**Table 3:** Binomial logistic regression model examining the determinants of M*d*SA^a^ and SS^b^

Factors	Moderate to severe anaemia			Severe stunting		
	Odds Ratio (OR)	95%(CI)	*P*-value	Odds Ratio (OR)	95%(CI)	*P*-value
Age			**0.023**			0.105
0.1.-1.0 year	1.69	1.02–2.81	**0.042**	0.56	0.31–0.98	**0.043**
1.1–2.0 years	1.94	1.19–3.17	**0.008**	0.98	0.54–1.52	0.712
^c^Male	1.02	0.68	1.52	1.34	0.86–2.09	0.196
Site			0.572			**< 0.001**
Dibanda	0.77	0.437–1.37	0.377	0.29	0.13–0.63	**0.001**
Ekona	1.02	0.64–1.63	0.923	1.23	0.76–2.00	0.394
Educational level			**< 0.001**			0.634
No formal	32.84	13.69–78.76	**< 0001**	0.73	0.28–1.88	0.513
Primary	2.56	1.11–5.91	**0.028**	0.70	0.27–1.79	0,451
Secondary	1.20	0.51–2.84	0.673	0.56	0.22–1.42	0.221
Feeding habit			0.111			0.991
NBF	1.55	0.91–2.64	0.109	0.96	0.54–1.72	0.892
EBF	1.55	0.99–2.54	0.086	0.99	0.57–1.72	0.966
^d^ Microcytosis	1.08	0.69–1.69	0.735	1.28	0.75–2.16	0.365
^e^ Severe stunting	1.81	1.04–3.14	**0.035**	–	–	–
^f^ Moderate to severe anaemia				1.77	1.04–3.01	**0.035**

^a^ Moderate to severe anaemia (M*d*SA) = children with Hb ≤ 10 g/dL

^b^ Severe stunting (SS) = children with a HA z-score of < −3

^c^ Male = 1 Female = 0

^d^ Microcytosis (MCV < 67 fL in children under 2 years of age and < 73 fL in children 2 to 5 years of age) = 1 Normal = 2

^e^ SS = 1 Normal = 0

^f^ M*d*SA = 1 Normal = 0

*P*- values in bold are statistically significant

## Discussion

The indirect effects of armed conflict are not limited to inadequate and unsafe living conditions, destruction of health, education, and economic infrastructure, but it is also a significant social determinant of child health [5]. We determined the prevalence of some of the health challenges such as moderate to severe anaemia and severe stunting facing children less than 3 years of age living in conflict-hit communities of Dibanda, Ekona and Muea located at the foot of Mount Cameroon.

The overall prevalence of anaemia of 84.0% demonstrate anaemia is a severe public health problem (prevalence ≥40%) in these communities according to the WHO classification [27]. This prevalence is more than twice the reported prevalence of anaemia in children in the area following malaria and anaemia intervention studies in 2013 [10]. In addition, it is higher than the 59.7% reported in pre-school children in Gaza Strip-Palestine [29] and the 54% reported in internally displaced children in Edo-Nigeria [30]. While the prevalence of malaria parasite in this population is lower than previously reported in children < than 5 years [10, 31, 32], that of anaemia has experienced an increase. The surge in prevalence of anaemia which is an important outcome indicator of poor nutrition and health may reflect the living conditions, poverty and increase in food insecurity in essentially farming communities that are unable to cultivate or harvest their produce due to insecurity in the region.

Children significantly affected by the burden of anaemia include the 0.1–1.0-year-old (88.3%), males (87.0%) and those whose parents had no formal education (98.2%). Similar categories of children have been identified in children < 2 years in Northeast Ethiopia [33] even though the prevalence of anaemia in these groups far exceeds the latter and the nationwide burden of anaemia in the same age [9]. This severe burden of anaemia in children 0.1–1.0 year may be attributed to the low practice of exclusive breast feeding (17.5%) and high practice of mixed feeding (62.1%) in a population where replacing breastmilk with iron fortified complementary feeding is actually a challenge in terms of quantity, quality and food insecurity as a result of the conflict. The high prevalence of anaemia observed in males is not unusual but may be related to their faster growth and demand for iron that cannot be met given the circumstances, while the highest occurrence of anaemia in children whose parents had no formal education could be a reflection of the poor socio-economic stability in this farming population.

Majority of the children had microcytic anaemia (59.3%) that occurred abundantly in males (64.6%), those undernourished (64.3%) especially the stunted (66.2%) and those whose parents had primary level of education (63.0%). Microcytic anaemia result from defective synthesis of haemoglobin causing a reduction in its mean corpuscular volume and the most common causes include iron deficiency anaemia, anaemia of chronic disease, α- thalassemias and sideroblastic anaemia [34–36]. The high proportion of microcytic anaemia which is probably linked to the occurrence of microcytosis (70.9%) suggests iron deficiency is the main cause of anaemia in the population. Iron deficiency anaemia, a main cause of microcytic anaemia in children [37] is a common nutritional problem whose occurrence may be exacerbated in conditions of nutritional inadequacy as evident in those undernourished due to food insecurity in conflict-stricken zones.

Observation from the study revealed moderate anaemia as the most common (52.4%) form of anaemia unlike the pre-conflict burden in the region that demonstrated a higher occurrence of mild anaemia [10]. The prevalence of moderate to severe anaemia (56.1%) is higher than the 50.2% observed in children < 2 years in Burkina Faso [38] and the ≤38.7% observed in children < 3 years in Nepal [39]. The high burden of moderate to severe anaemia probably reflects the burden of disease in the population. While the burden of disease may increase during conflict, access to health care becomes increasingly difficult especially in pregnant women displaced by the conflict who are unable to receive prenatal care and the new-born who are less likely to receive vaccination with dire consequences. The contextual determinants of moderate to severe anaemia in this conflict-hit area include age (< 2 years), educational level of parent/caregiver (no formal and primary education) and severe stunting. This finding may be inadequate since Ngnie-Teta et al. [40] in addition to the determinants enumerated, identified incomplete immunization, recent infection, absence of bed nets, as risk factors for moderate to severe anaemia which are potential determinants in this at-risk population that were not evaluated. However, the burden of moderate to severe anaemia in the identified groups especially in those with severe stunting (64.6%) warrants immediate intervention to alleviate their dire health.

Findings from the study revealed undernutrition is a public health problem in the study population. The high prevalence (range = 30–39%) of stunting (31.3%), medium prevalence (10–19%) of underweight (13.1%) and poor prevalence (5–9%) of wasting (6.3%) is comparable to the prevalence of stunting (31%), underweight (14%) and wasting (6%) observed in children < 2 years in Batouri, East Region, Cameroon, the area with the highest percentage of stunting [41, 42]. The prevalence of stunting is higher than the 16.4% observed in Banja village and 12.8% in Yaounde, Cameroon [42, 43] and lower than the 42% observed in children < 3 years in northern province of Rwanda [44]. The increase in linear growth retardation when compared with pre conflict prevalence of 19.7% in the Mount Cameroon area [22] likely demonstrates the cumulative effect of the negative impact of starvation and lack of adequate food intake consequential from consistent violence that has forced the farming communities to abandon their livelihood.

Of grave public health significance is the very high prevalence of stunting (≥40%) in children of Ekona (41%) and those whose parent had tertiary education (40%) while that of wasting (10–14%) occurred in non-breast-fed children (10.7%). While site specific differences do exist, Ekona is among the hardest hit zone in the area [45]. The constant movement of people in search of shelter and security has worsened their living conditions leaving regularly consumed crops like corn, cassava, potatoes unharvested in farmlands. Most parents/caregivers in these areas are farmers with primary or no formal education while the few with tertiary education are employed by the state in state institutions. The high occurrence of stunting in children whose parent had tertiary education is unusual when several authors have reported the contrary [46–48]. This probably highlights the precarious conditions in conflict-hit areas where breakdown in the farm to market channels severely affects even the economically viable. The importance of exclusive breast feeding whose practice is low (20.6%) in the population and includes the prevention of growth faltering [49], is probably demonstrated in the serious occurrence of wasting in the non-breast-fed infant. However, the low practice of exclusive breast feeding may be attributed to the lack of adequate information being given to the mothers, level of education and inability to produce enough breast milk to satisfy the infant due to the apparent living conditions.

The prevalence of severe stunting (17.1%) is comparable to the 16.4% reported in Nigeria [50], lower than the 29.1% reported in Northern Cameroon [51] and the 25.1% reported in Wardha, Central India [17] but higher than the 10.2% observed in Nepal [52]. The nature of diet of children in the Mount Cameroon area is similar but the access to food may vary currently depending on the intensity of the violence in the area. The significantly lower risk of severe stunting observed in children living in Dibanda may be linked to the relatively safer living conditions and better access to food when compared with the other study sites. In line with other studies [48, 52, 53], children < 1 year were less likely to be severely stunted. This attests to the chronic nature of stunting hence the effect of the inappropriate weaning foods in terms of quality and quantity is only apparent with increasing age.

Moderate to severe anaemia was the only significant determinant of severe stunting as findings from the study revealed a significantly higher co-occurrence of stunting and anaemia (33.2%). This prevalence is higher than the 23.9% reported in young children in Ethiopia [54]. The co-morbid condition of stunting and anaemia has been reported to be common especially among more disadvantaged children in low- and middle-income countries [55], a reflection of the condition of the study population. In view of the fact that household food insecurity has been linked to stunting and anaemia [56], the severe stunting in moderate to severe anaemic children may be attributed to the low consumption of meat, vitamin A rich fruit and vegetables diets and reduced meal frequency. Furthermore, untreated infections may also be a contributing factor due to the breakdown of health system in the area.

A limitation to this study is the cross-sectional nature of the design that could only capture the burden at a point in time and the limited number of measurable covariates evaluated. Notwithstanding, the findings bring to light some of the challenges facing a vulnerable group of children in a conflict-hit area. Given the security challenges and further degeneration in violence in the region, the health challenges of these vulnerable population would have deteriorated even further requiring immediate intervention.

## Conclusions

The determinants of moderate to severe anaemia are age < 2 years, educational level of parents /caregivers and severe stunting while the determinant of severe stunting is moderate to severe anaemia. Moderate to severe anaemia, severe stunting and wasting especially in children not breastfed at all are public health challenges in the conflict-hit zones in the Mount Cameroon area that requires immediate attention to improve upon the health of the children. The varying intensity of the conflict by site has negative influence on the nutritional status of the children with very high prevalence of stunting in children in the hardest hit zone of Ekona. While there is a need for targeted intervention to control anaemia as well as increase awareness of exclusive breast feeding in conflict hit areas, a peaceful resolution of the conflict is necessary to alleviate the health of the children.

## Supplementary information

**Additional file 1.** Mean (SD) clinical and laboratory characteristics of participants by sex and age. This file shows children 0.1–1.0-year-old had highest mean WBC and RBC counts, those 1.1–2.0 years had highest RDW-CV while those 2.1–3.0 year’s old had highest temperature, MCV, MCH and lowest platelet counts. The differences were statistically significant.

**Additional file 2.** Prevalence of undernutrition and its forms by demographic and clinical factors. The file shows significantly higher prevalence of undernutrition (45.8%) in children from Ekona community while underweight was significantly higher in MP negative children (15.1%).

**Additional file 3.** This is the manuscript data.

## Data Availability

All datasets generated and analysed during the current study are presented in the paper and supporting information files.

## Abbreviations

ANOVA: Analysis of variance
AMP: Asymptomatic malaria parasitaemia
CI: 95% confidence interval
EBF: Exclusively breastfeeding
Hct: Haematocrit
Hb: Haemoglobin
HA: Height-for-age
MCH: Mean corpuscular haemoglobin
MCHC: Mean corpuscular Hb concentration
MCV: Mean corpuscular volume
MF: Mixed feeding
M*d*SA: Moderate-to-severe anaemia
NBF: No breast feeding
OR: Odd ratios
RBC: Red blood cell
RDW-CV: Red cell distribution width coefficient of variation
SS: Severe stunting
SD: Standard deviation
WA: Weight-for-age
WH: Weight-for-height
WBC: White blood cell
WHO: World health organisation
