# Supplementary material for: Burden of moderate to severe anaemia and severe stunting in children < 3 years in conflict-hit Mount Cameroon: a community based descriptive cross-sectional study
Source: BMC Pediatr. 2020 Aug 24;20:396. doi: 10.1186/s12887-020-02296-2 (PMC7445924; doi:10.1186/s12887-020-02296-2)
Supplement: Supplementary file 1 — Additional file 1. Mean (SD) clinical and laboratory characteristics of participants by sex and age. This file shows children 0.1–1.0-year-old had highest mean WBC and RBC counts, those 1.1–2.0 years had highest RDW-CV while those 2.1–3.0 year’s old had highest temperature, MCV, MCH and lowest platelet counts. The differences were statistically significant. [file 12887_2020_2296_MOESM1_ESM.docx]

**Additional file 1. Mean (SD) clinical and laboratory characteristics of participants by sex and age.**

| Parameter | Sex | | Test | Age group in years | | | Overall | Test |
| --- | --- | --- | --- | --- | --- | --- | --- | --- |
|  | Male | Female |  | 0.1-1.0 | 1.1-2.0 | 2.1-3.0 |  |  |
| Age | 1.7 (0.9) | 1.8 (0.9) | 0.70 | 0.7 (0.3) | 1.7 (0.3) | 2.8 (0.3) | 1.8 (0.1) |  |
| Height in cm | 79.8 (13.1) | 80.0 (13.3) | 0.865 | 69.8 (12.5)^a^ | 80.5 (10.9)^b^ | 88.9 (8.4)^c^ | 79.9 (13.2) | **<0.001** |
| Weight in kg | 11.3 (3.3) | 11.2 (3.8) | 0.61 | 9.0 (3.6) ^a^ | 11.1 (2.9) ^b^ | 13.4 (2.8) ^c^ | 11.2 (3.6) | **<0.001** |
| Temperature in ^o^C | 36.5 (0.6) | 36.5 (0.6) | 0.252 | 36.5 (0.5) ^ad^ | 36.4 (0.6) ^b^ | 36.6 (0.7)^cd^ | 36.5 (0.6) | **<0.001** |
| GMPD (n) | 684 (98) | 596 (93) | 0.518 | 681 (66) | 713 (59) | 545 (66) | 640 (191) | 0.539 |
| Hb in g/dL | 9.5 (1.5) | 9.4 (1.4) | **0.018** | 9.6 (1.5) | 9.6 (1.4) | 9.7 (1.6) | 9.6 (1.5) | 0.700 |
| Hct in % | 25.6 (4.2) | 26.6 (4.0) | **0.002** | 26.1 (4.1) | 26.0 (4.1) | 26.2 (4.2) | 26.1 (4.1) | 0.880 |
| WBC x 10 ^9^/L | 8.7 (2.4) | 8.6 (2.4) | 0.838 | 9.0 (2.4) ^ac^ | 8.8 (2.4) ^c^ | 8.2 (2.3) ^b^ | 8.6 (2.4) | **0.001** |
| RBC x 10^12^/L | 4.0 (0.9) | 4.1 (1.0) | 0.**031** | 4.2 (1.1) ^a^ | 4.1 (1.0) | 3.9 (0.8) ^b^ | 4.0 (1.0) | **0.012** |
| MCV in fL | 66.0 (7.5) | 67.1 (6.9) | 0.065 | 65.3 (8.4) ^ac^ | 66.1 (7.3) ^c^ | 68.3 (5.6) ^b^ | 66.6 (7.2) | **<0.001** |
| MCH in pg | 24.4 (3.4) | 24.4 (4.3) | 0.911 | 23.9 (4.5) ^ac^ | 24.0 (3.5) ^c^ | 25.2 (3.4) ^b^ | 24.4 (3.9) | **0.001** |
| MCHC in g/L | 37.1 (3.9) | 36.4 (5.4) | 0.058 | 36.8 (5.5) | 36.5 (4.3) | 37.0 (4.4) | 36.7 (4.7) | 0.466 |
| Platelet/L | 254.4 (124.3) | 266.4 (152.9) | 0.275 | 271.4 (132.4) ^ac^ | 271.5 (141.6) ^c^ | 239.1 (141.9) ^b^ | 260.4 (139.5) | **0.020** |
| RDW-CV% | 14.0 (6.5) | 13.2 (1.9) | 0.055 | 13.7 (2.0) | 14.2 (7.7)^a^ | 12.9 (1.7)^b^ | 13.6 (4.8) | **0.016** |

Post Hoc Test shows means with different letters are significantly different. Hct = haematocrit, Hb = haemoglobin, MCH = mean corpuscular haemoglobin, MCHC = mean corpuscular Hb concentration, MCV = mean corpuscular volume, RBC = red blood cell, RDW-CV = red cell distribution width coefficient of variation.
