# Supplementary material for: Burden of moderate to severe anaemia and severe stunting in children < 3 years in conflict-hit Mount Cameroon: a community based descriptive cross-sectional study
Source: BMC Pediatr. 2020 Aug 24;20:396. doi: 10.1186/s12887-020-02296-2 (PMC7445924; doi:10.1186/s12887-020-02296-2)
Supplement: Supplementary file 2 — Additional file 2. Prevalence of undernutrition and its forms by demographic and clinical factors. The file shows significantly higher prevalence of undernutrition (45.8%) in children from Ekona community while underweight was significantly higher in MP negative children (15.1%). [file 12887_2020_2296_MOESM2_ESM.docx]

**Additional file 2. Prevalence of undernutrition and its forms by demographic and clinical factors.**

| Characteristic | Category | N | Prevalence of undernutrition (n) | P value | Prevalence of the different forms of undernutrition (n) | | | | | |
| --- | --- | --- | --- | --- | --- | --- | --- | --- | --- | --- |
|  |  |  |  |  | Stunting | P value | Under  weight | P value | Wasting | P value |
| All | All | 649 | 38.4 (249) |  | 31.3 (203) |  | 13.1 (85) |  | 6.3 (41) |  |
| Socio-demographic factors | | | | | | | | | | |
| Age group in years | 0.1 1.0 | 206 | 35.5 (73) | 0.571 | 27.2 (56) | 0.308 | 13.1 (27) | 0.689 | 7.3 (15) | 0.789 |
|  | 1.1 – 2.0 | 222 | 40.1 (89) |  | 33.3 (74) |  | 11.7 (26) |  | 5.9 (13) |  |
|  | 2.1 – 3.0 | 221 | 39.4 (87) |  | 33.0 (73) |  | 14.5 (32) |  | 5.9 (19) |  |
| Sex | Female | 327 | 35.5 (116) | 0.127 | 29.1 (95) | 0.218 | 12.2 (40) | 0.511 | 5.2 (17) | 0.238 |
|  | Male | 322 | 41.3 (133) |  | 33.5 (108) |  | 14.0 (45) |  | 7.5 (24) |  |
| Site | Dibanda | 161 | 24.8 (40) | **<0.001** | 18.6 (30) | **<0.001** | 12.4 (20) | 0.634 | 5.6 (9) | 0.079 |
|  | Ekona | 273 | 45.8 (125) |  | 41.0 (112) |  | 12.1 (33) |  | 4.4 (12) |  |
|  | Muea | 215 | 39.1 (84) |  | 28.4 (61) |  | 14.9 (32) |  | 9.3 (20) |  |
| Educational level of parent/  caregiver* | No formal | 273 | 38.5 (105) | 0.331 | 30.8 (84) | 0.557 | 15.8 (43) | 0.215 | 8.4 (23) | 0.228 |
|  | Primary | 154 | 37.7 (58) |  | 30.5 (47) |  | 13.6 (21) |  | 5.8 (9) |  |
|  | Secondary | 166 | 34.3 (57) |  | 28.3 (47) |  | 9.0 (15) |  | 4.2 (7) |  |
|  | Tertiary | 40 | 50.0 (20) |  | 40.0 (16) |  | 10.0 (4) |  | 2.5 (1) |  |
| Infant feeding habit | NBF | 122 | 41.0 (50) | 0.612 | 30.3 (37) | 0.762 | 13.9 (17) | 0.594 | 10,7 (13) | 0.092 |
|  | EBF | 134 | 35.1 (47) |  | 29.1 (39) |  | 10.4 (14) |  | 5.2 (7) |  |
|  | MF | 393 | 38.7 (152) |  | 32.3 (127) |  | 13.7 (54) |  | 5.3 (21) |  |
| Clinical factor | | | | | | | | | | |
| Febrile status | Afebrile | 613 | 38.3 (235) | 0.947 | 31.2 (191) | 0.784 | 19.4 (7) | 0.245 | 5.6 (2) | 0.847 |
|  | Febrile | 36 | 38.9 (14) |  | 33.3 (12) |  | 12.7 (78) |  | 6.4 (39) |  |
| Malaria parasite status | Positive | 191 | 38.2 (73) | 0.096 | 32.5 (62) | 0.675 | 8.4 (16) | **0.021** | 5.2 (10) | 0.464 |
|  | Negative | 458 | 38.4 (176) |  | 30.8 (141) |  | 15.1 (69) |  | 6.8 (31) |  |
| Anaemic | Yes | 545 | 39.8 (217) | 0.082 | 33.2 (181) | **0.015** | 13.6 (74) | 0.406 | 7.0 (38) | 0.116 |
|  | No | 104 | 30.8 (32) |  | 21.2 (22) |  | 10.6 (11) |  | 2.9 (3) |  |

P values in bold are statistically significant. EBF= exclusive breastfeeding, MF = mixed feeding, NBF = no breast feeding.
